# The Diseases of the Lungs

**Published:** 1898-12

**Authors:** 


**The Diseases of the Lungs.** By JAMES KINGSTON FOWLER, M.D., and RICKMAN JOHN GODLEE. Pp. xv., 715. London: Longmans, Green, and Co. 1898.

The combined work of two well-known authorities—the one a physician the other a surgeon—endeavours to present a continuous picture of the medical and surgical aspects of pulmonary disease. An anatomical chapter of 49 pages gives an excellent outline of the structures concerned, and is exceedingly well illustrated. A short chapter on physical diagnosis follows, and we notice that the combined method of auscultatory percussion is beginning to be recognised as of service in defining the outlines of hollow cavities in addition to its use in determining the outlines of the heart.

Nine chapters are devoted to diseases of the trachea and bronchi; eight following describe the varietes of pneumonia, in which the bacterial origin in many cases is well recognised and the possibility of contagion admitted. The treatment advised by Dr. Fowler is not too active: he does not advise the use of any antipyretic drugs, he does not give quinine even in the hyperpyretic cases, and systematic treatment by digitalis in full doses is not mentioned. As regards the treatment by serum, he states that no ill effects have followed its use, but he considers it premature to advise a general employment of the antitoxin treatment. Although poultices are somewhat out of fashion, the author confesses a preference for leeches and poulticing over the more modern ice-bag.

The subject of pulmonary tuberculosis occupies ten chapters: the author has for many years urged that when tubercle is present in the lungs the most appropriate name for the disease is pulmonary tuberculosis, and that the term phthisis should be to a great extent abolished; he also gets rid of that most misleading phrase "the stages of phthisis." The definition is as follows: "a disease of the lungs due to the presence of a specific organism, the bacillus tuberculosis"; everything circles around the bacillus as the *fons et origo mali*, and the importance of catarrh in the etiology of the disease has been much exaggerated. As regards the use of tuberculin, the author states that experience is not yet sufficient to justify any statement of opinion as to its value. Serum treatment, nucleins, and antiseptic treatment do not find any commendation; of tonic remedies, "the most valuable are cod-liver oil, the hypophosphites, arsenic, quinine, nux vomica, mineral acids, and vegetable bitters such as gentian." Climatic treatment, more particularly that at high altitudes, is considered to be far more valuable than that to be obtained in any other way. The surgical treatment of pulmonary cavities forms an interesting chapter by Mr. Godlee, who comments upon the fact that so little enthusiasm has been called forth on a question on which it would be rash to prophesy that there is not a future. We have often wondered why it is that pulmonary *cavities* alone should be considered as requiring treatment, and have made many attempts to deal with masses of *consolidated lung* by local injection into the focus of the tubercular disease: the effects of such attempts are recorded elsewhere, but intra-pulmonary antiseptic injections have not met with a very favourable reception either by the profession or the patients.

Chapter LXII. directs attention to the significance of pain in diseases of the lungs and pleura, and quotes the conclusions of Dr. Head on the subject of referred pain. A chapter on the changes in joints and bones associated with intra-thoracic diseases, and one on clubbing of the fingers and toes, conclude a volume which deserves to be looked upon as the most recent and trustworthy monograph on chest diseases in the English language.

